# A rare case report of an acquired aortopulmonary artery fistula after Bentall procedure: multimodality imaging approach may be the key?

**DOI:** 10.1093/ehjcr/ytae236

**Published:** 2024-05-07

**Authors:** Monica Barki, Stefania Sacchi, Cecilia Marcolin, Silvia Ajello, Anna Mara Scandroglio

**Affiliations:** Cardiac Intensive Care Unit, San Raffaele University Hospital, Via Olgettina 60, 20132 Milan, Italy; Cardiac Intensive Care Unit, San Raffaele University Hospital, Via Olgettina 60, 20132 Milan, Italy; Cardiac Intensive Care Unit, San Raffaele University Hospital, Via Olgettina 60, 20132 Milan, Italy; Cardiac Intensive Care Unit, San Raffaele University Hospital, Via Olgettina 60, 20132 Milan, Italy; Cardiac Intensive Care Unit, San Raffaele University Hospital, Via Olgettina 60, 20132 Milan, Italy

**Keywords:** Acute respiratory failure, Aortopulmonary fistula, Multimodality imaging approach, Case report

## Abstract

**Background:**

The acquired communication between the aorta and the pulmonary artery is a rare and potentially life-threatening condition. Its diagnosis is challenging and may require a multimodality imaging approach.

**Case summary:**

A 67-year-old Caucasian man, admitted for acute respiratory failure unresponsive to medical therapy and non-invasive ventilation, was diagnosed with an aortopulmonary fistula (APF) complicating a pseudoaneurysm of the aortic root. This condition developed after Bentall cardiac surgery, which entailed the use of a straight Dacron aortic graft coupled with a mechanical prosthesis. A multimodal imaging approach, combining echocardiography and computed tomography angiography, was diagnostic and supported the development of a surgical treatment strategy. The patient underwent successful surgical closure of the APF and correction of the aortic pseudoaneurysm.

**Discussion:**

Aortopulmonary fistula can result in rapid clinical deterioration if left untreated. The combination of echocardiography and computed tomography angiography techniques allowed for the diagnosis and surgical correction of the APF.

**Learning points**

- An aortopulmonary fistula (APF) can lead to rapid clinical deterioration, with a bleak prognosis for the patient if left untreated.
- A multimodality imaging approach, incorporating echocardiography and computed tomography angiography, may prove pivotal in diagnosing APF and directing the surgical strategy for correcting the defect.

## Introduction

The acquired communication between the aorta and the pulmonary artery has been reported as a rare and potentially life-threatening complication of long-standing aortic aneurism and aortic dissection.^1–3^ Case reports have shown its associations with infective endocarditis and valvular surgery. However, the majority of aortopulmonary fistula (APF) cases has been diagnosed post-mortem and extremely rarely in patients who are still alive.^4^ Thus, the diagnosis of this entity can be challenging, and a combination of newer multimodality techniques such as computed tomography angiography can help overcome the limitations of traditional imaging modalities like echocardiography, assisting in the assessment of such complex cases. In the following case report, we discuss the rare occurrence of an APF formation following a previous mechanic Bentall procedure for aortic root replacement.

## Summary figure

**Table:** 

TIME	EVENT
**DAY 0**	A 67-year-old man with a history of mechanic Bentall procedure 19 years before presents to the emergency department with acute respiratory distress. Transthoracic echocardiography shows biventricular moderate dysfunction with a normally functioning mechanical aortic valve. Diuretics and non-invasive ventilation are started. Blood cultures are collected.
**DAY 1**	The patient is intubated due to persistent severe respiratory failure despite non-invasive ventilation, diuretics, and inotropes.
**DAY 4**	Transoesophageal echocardiography demonstrates a voluminous pseudoaneurysm of the aortic root and a direct communication between it and the pulmonary artery with a massive left-to-right shunt causing pulmonary hyper flow. Computed tomography angiography shows a supravalvular aortic pseudoaneurysm (max dimension 5 × 3 × 4.6 cm) communicating with the right ventricular outflow tract with the evidence of an aortopulmonary fistula (diameter 10 mm) associated with a left-to-right holosystolic shunt leading to dilated pulmonary arteries and pulmonary hyper flow. Blood cultures are negative. A Heart Team discussion is conducted.
**DAY 5**	The patient undergoes cardiac surgery, including surgical correction of the aortic pseudoaneurysm with a tubular prosthesis, composite graft and direct reimplantation of the left main coronary artery into an interposition graft (Cabrol graft–left main anastomosis), and heterologous pericardial patch closure of the aortopulmonary fistula.
**DAY 6**	An improvement in respiratory exchanges is observed, resulting in extubation from the orotracheal tube, along with an improvement in haemodynamic status and the recovery of cardiac function, leading to the weaning from inotropic support.
**1 MONTH**	The patient is recovering well.

## Case presentation

**Figure 1:**
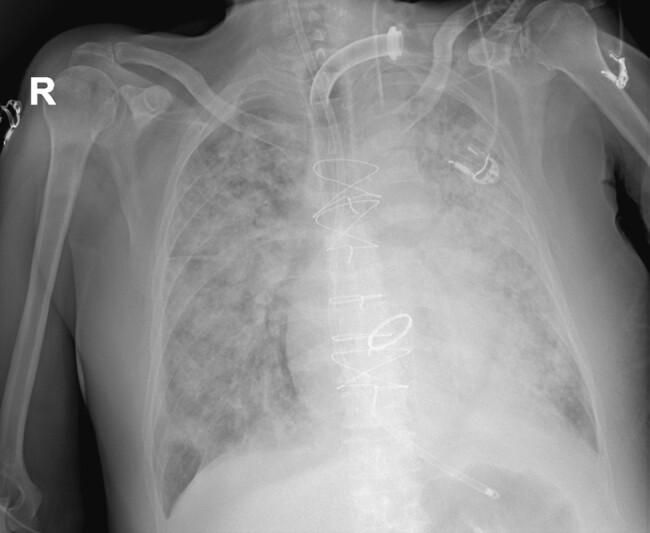
Chest X-ray showing acute lung injury with bilateral involvement (acute respiratory distress syndrome—ARDS) in patient mechanically ventilated through the tracheostomy tube.

A 67-year-old man with history of mechanic Bentall procedure 19 years earlier presented to the emergency department with acute respiratory distress. The examination revealed a severe increase in blood pressure (180/100 mmHg), along with severe respiratory failure and a heart rate of 130 b.p.m. Bilateral lung crackles were detected, without significant bipedal oedema. An electrocardiogram showed sinus tachycardia. Chest X-ray revealed cardiomegaly and bilateral pulmonary congestion. Laboratory tests showed normal electrolytes, mildly impaired renal function (creatinine 1.3 mg/dL), and a complete blood count (white cells 11 × 10^9^/L, red cells 4.7 ×10^12^/L, platelet 250 × 10^9^/L). Blood cultures were negative. After the initial conservative management with diuretic and non-invasive ventilation failed, the patient was sedated and intubated due to the persistent severe respiratory failure (*Figure 1*).

Transthoracic echocardiography (TTE) revealed moderate biventricular dysfunction, a well-functioning mechanical aortic valve (mean gradient 5.5 mmHg, maximum velocity 1.7 m/s, Doppler velocity index 0.7), and a ruptured saccular aneurysm with a large, continuous, and holosystolic flow in the main pulmonary artery, originating in the aorta, and highly suspicious of an APF.

**Figure 2:**
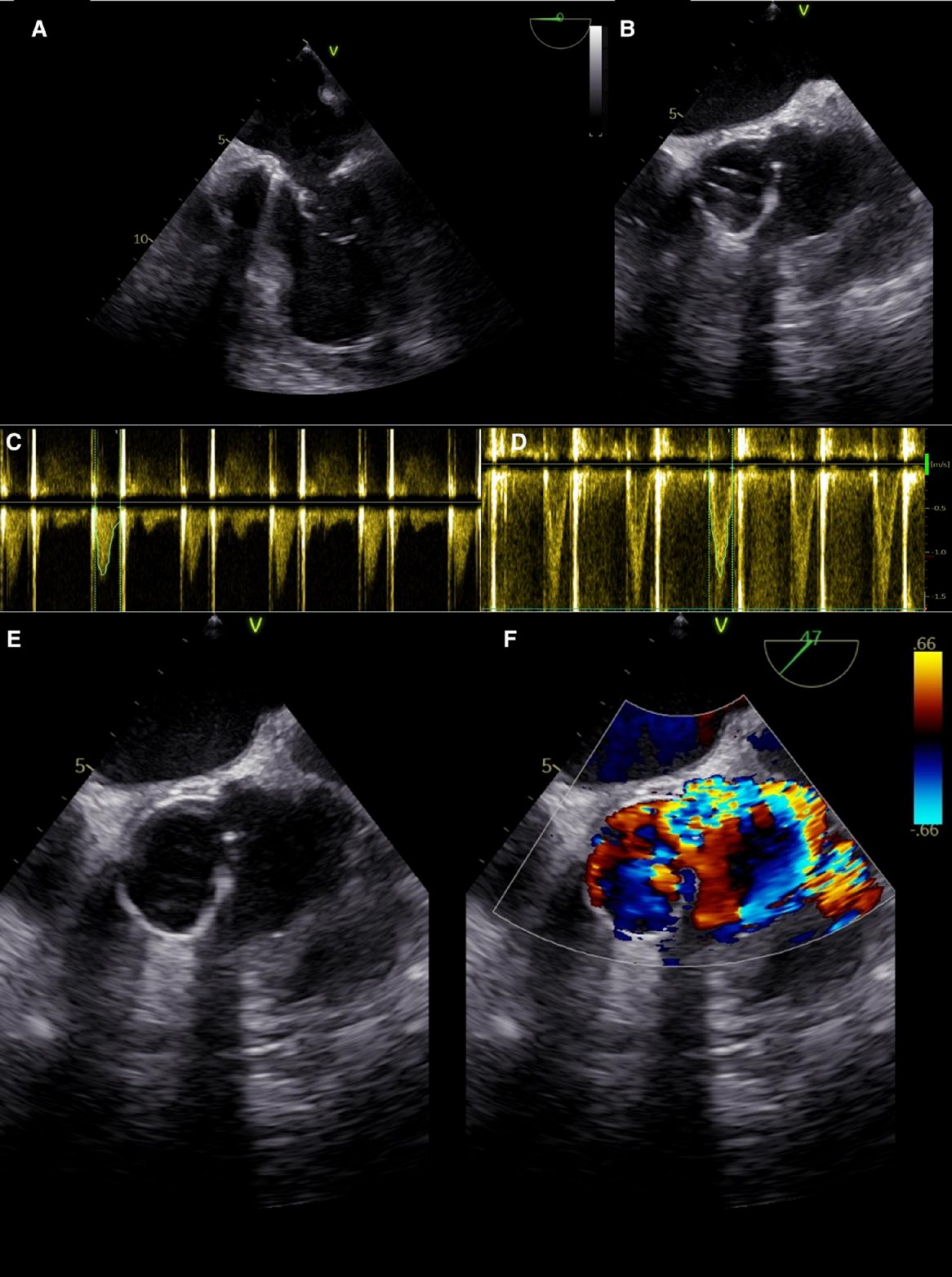
Transthoracic echocardiography and transoesophageal echocardiography images. Transoesophageal bicommisural view (*A*) demonstrating a mild to moderate dilation of the left ventricle. Transoesophageal short-axis view (*B*) showing the mechanical prosthetic aortic valve and the pseudoaneurysm of the aortic root. Continuous and pulsed wave Doppler at transthoracic echocardiography (*C–D*) demonstrating a normally functioning mechanical aortic valve (mean gradient 5.5 mmHg, max. velocity 1.7 m/s, DVI 0.7). Zoomed short-axis transoesophageal view (*E–F*) exhibiting the voluminous pseudoaneurysm with the colour Doppler demonstrating the communication of the pseudoaneurysm with the pulmonary artery with a left-to-right shunt.

Further assessment with transoesophageal echocardiography (TOE) confirmed the presence of a voluminous pseudoaneurysm of the aortic root communicating with the left coronary sinus and with the pulmonary artery causing an APF. Doppler assessment confirmed continuous forward flow from the ascending aorta to the pulmonary trunk (*Figure 2*).

**Figure 3:**
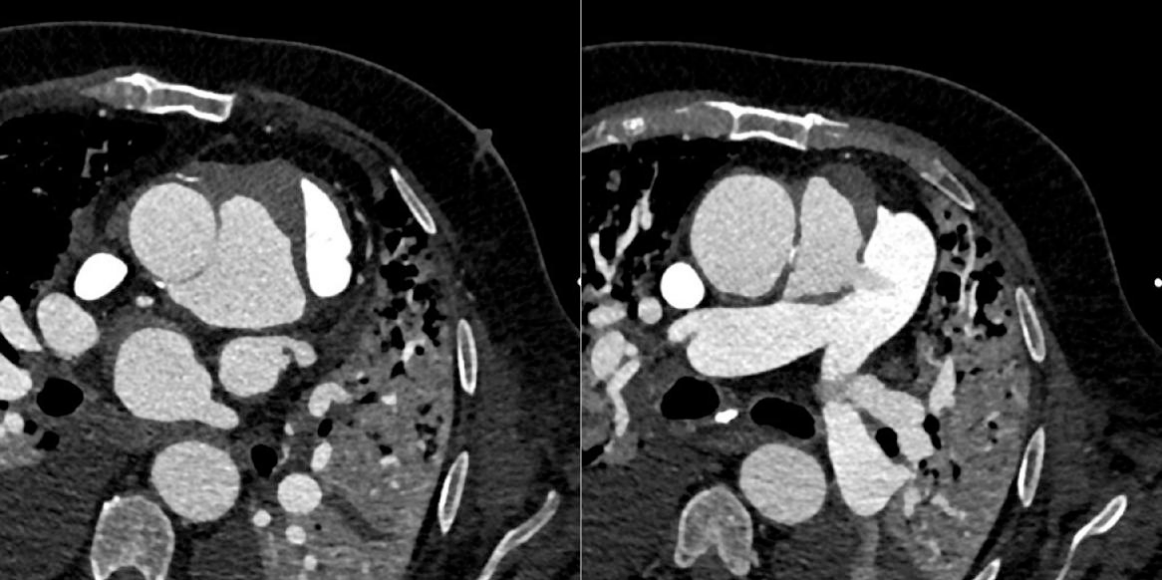
Contrast computed tomography angiography images in oblique axial reconstruction demonstrating the voluminous pseudoaneurysm of the aortic root (left) and the aortopulmonary fistula (right).

Computed tomography angiography (CTA) demonstrated normal coronary arteries and a corrected excursion of the mechanic leaflet of the aortic valve. The presence of a broad supravalvular aortic pseudoaneurysm (maximum dimension 5 × 3 × 4.6 cm) communicating with the right ventricular outflow tract (RVOT) was confirmed, along with the evidence of an APF located just anterior to the origin of the left main coronary artery and extending just before the junction of the main pulmonary artery with the right pulmonary artery (diameter 10 mm). The fistula was associated with a left-to-right holosystolic shunt resulting in dilated pulmonary arteries and pulmonary hyper flow (*Figure 3*).

The patient underwent surgical correction of the aortic pseudoaneurysm with a tubular prosthesis, a composite Dacron graft, direct reimplantation of left main coronary artery into the interposition graft (Cabrol graft–left main anastomosis), and closure of the APF with a heterologous pericardial patch.

**Figure 4:**
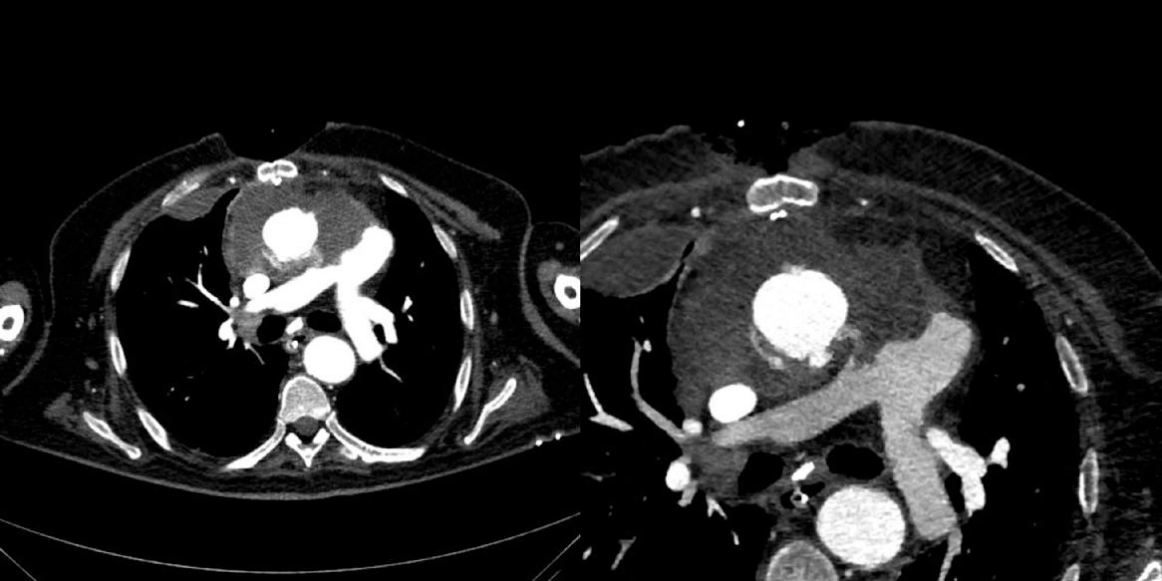
Post-operative contrast computed tomography angiography images in oblique axial reconstruction demonstrating the complete resolution of the aortopulmonary fistula (APF).

He experienced improvement in respiratory exchanges with no residual APF and left-to-right shunt and normalized pulmonary flow, leading to extubation from the orotracheal tube. Additionally, there was improvement in haemodynamic status and recovery of cardiac function, resulting in the discontinuation of inotropic support (*Figure 4*).

## Discussion

Aortopulmonary fistula represents an extremely rare but potentially lethal condition.^5^ With an estimated prevalence of 4% in post-mortem case, APF can be spontaneous (degenerative atherosclerosis) or secondary to aortitis, thoracic trauma, dissecting aortic aneurysm, thoracic aortic or sinus of Valsalva aneurysm rupture, infective endocarditis, or as a complication of procedures performed on the aorta or pulmonic valve.^1,2^

In our patient, the pseudoaneurysm of the aortic root was considered as a chronic complication of the previous Bentall surgery, whereas the APF as an acute-on-chronic complication was deemed responsible for patient’s respiratory failure and haemodynamic instability. Unfortunately, we were unable to document and date the occurrence of the pseudoaneurysm, as we lacked available patient data regarding the post-cardiac surgery follow-up, which was reported as regular. Upon admission, the blood count was within range, and serial blood cultures were negative, leading us to exclude an underlying acute infective endocarditis process.

The Bentall procedure involves a straight Dacron aortic graft coupled with a mechanical prosthesis. These techniques has shown favourable results with encouraging 30-day mortality and 5 years survival rates.^6,7^ However, major complications may still occur. In this setting, pseudoaneurysms originating from dehiscence of suture lines are one of the most feared and lethal complication, with a highly variable and anecdotic incidence.^8,9^ They evolve insidiously and can cause life-threatening conditions such as compression and fistulation to other structures.^8,9^ In our case, the pseudoaneurysm had already caused the fistulation to the pulmonary artery, resulting in an APF and pulmonary hyper flow.

Cases of acquired APF, following cardiac surgery or trauma, characterized by an insidious onset of heart failure that remained undiagnosed for months, have been previously reported.^10,11^ In our case, the presentation of APF was dramatic, with an acute onset of refractory respiratory failure. In any scenario, however, irrespective of the presenting symptoms, a multimodal cardiac imaging approach was instrumental in establishing the diagnosis of APF.

Imaging is the mainstay of APF diagnosis, with echocardiography often showing continuous flow from the aortic root to the pulmonary artery.^1,2,12^ In this case, the suspicion of APF arose from TTE, which revealed high-velocity continuous flow into the pulmonary artery. The diagnosis was subsequently confirmed by TOE and CTA. Specifically, CTA allowed for the precise localization of the communication site and the measurement of the APF’s dimensions together with a more comprehensive view of the entire aorta.^1,2,12^

An APF requires urgent treatment, typically involving an open procedure.^5,13,14^ In cases where this approach is contraindicated, an endovascular stent can be considered as a less invasive alternative.^5,13,14^ In this particular case, given the patient’s history of previous Bentall surgery, the presence of a concomitant aortic root pseudoaneurysm, and the low patient’s comorbidities, a surgical APF closure with heterologous pericardial patch was performed. Additionally, a complex surgical correction of the aortic pseudoaneurysm was carried out with tubular prosthesis and direct reimplantation of left main coronary artery. The patient underwent successful surgical treatment and had a favourable outcome.

In summary, our clinical case presented several challenging aspects. Firstly, the persistent respiratory failure, unresponsive to medical therapy and non-invasive ventilation, in the absence of pneumonia and severe cardiac disease, prompted us to investigate alternative less common causes of respiratory failure such as APF. Secondly, the use of multimodality imaging approach, combining echocardiography and computed tomography, played a pivotal role in the diagnosis of APF and in guiding the surgical plan. Finally, prompt surgical treatment was effective and resolved the APF, leading to the resolution of symptoms.

## Conclusion

This clinical case emphasizes how a rare condition, such as APF complicating a long-standing pseudoaneurysm of the aortic root after Bentall cardiac surgery, can result in rapid clinical and haemodynamic deterioration with a poor prognosis if left untreated. In this context, a multimodality imaging approach that included TEE, TOE, and CTA played a crucial role in identifying and locating the site of APF communication, as well as guiding the strategy for surgically correcting the defect.

## Data Availability

The data underlying this article are available in the article.

## Lead author biography

Stefania Sacchi graduated in Medicine and Surgery from San Raffaele University, Milan, Italy, in 2005. She specialized in arrhythmias during a fellowship at San Raffaele Hospital (2006–09). She completed her cardiology residency at the University of Florence (2009–14) and research fellowship in arrhythmias at the Texas Cardiac Arrhythmic Institute, Austin, Texas, USA, in 2012. She achieved a PhD in clinical sciences from the University of Florence (2014–17) and held roles including visiting researcher at Imperial College London (2015–16) and postdoctoral researcher in heart failure at IRCCS Multimedica, Milan, Italy (2017–18). She is currently a cardiologist in the Cardiac Intensive Care Unit at San Raffaele University Hospital, Milan, Italy.

**Consent:** The authors confirm that written consent for submission and publication of this case report, including images and associated text, has been obtained from the patient in accordance with the Committee on Publication Ethics (COPE) guidelines.

**Funding:** None declared.
